# Using Targeted Nano-Antibiotics to Improve Antibiotic Efficacy against *Staphylococcus aureus* Infections

**DOI:** 10.3390/antibiotics12061066

**Published:** 2023-06-16

**Authors:** Hung Le, Emmanuelle Dé, Didier Le Cerf, Carole Karakasyan

**Affiliations:** Sciences & Technic Faculty, Univ Rouen Normandie, INSA Rouen Normandie, CNRS, PBS UMR 6270, 76000 Rouen, France; hung.le1@univ-rouen.fr (H.L.); emmanuelle.de@univ-rouen.fr (E.D.); carole.karakasyan@univ-rouen.fr (C.K.)

**Keywords:** nano-antibiotic, targeted delivery, *S. aureus*, nano-medicine, enhanced pharmacokinetic

## Abstract

The poor bioavailability of antibiotics at infection sites is one of the leading causes of treatment failure and increased bacterial resistance. Therefore, developing novel, non-conventional antibiotic delivery strategies to deal with bacterial pathogens is essential. Here, we investigated the encapsulation of two fluoroquinolones, ciprofloxacin and levofloxacin, into polymer-based nano-carriers (nano-antibiotics), with the goal of increasing their local bioavailability at bacterial infection sites. The formulations were optimized to achieve maximal drug loading. The surfaces of nano-antibiotics were modified with anti-staphylococcal antibodies as ligand molecules to target *S. aureus* pathogens. The interaction of nano-antibiotics with the bacterial cells was investigated via fluorescent confocal microscopy. Conventional tests (MIC and MBC) were used to examine the antibacterial properties of nano-antibiotic formulations. Simultaneously, a bioluminescence assay model was employed, revealing the rapid and efficient assessment of the antibacterial potency of colloidal systems. In comparison to the free-form antibiotic, the targeted nano-antibiotic exhibited enhanced antimicrobial activity against both the planktonic and biofilm forms of *S. aureus*. Furthermore, our data suggested that the efficacy of a targeted nano-antibiotic treatment can be influenced by its antibiotic release profile.

## 1. Introduction

Antibiotic resistance presents one of the biggest threats to global health today. Despite continuous efforts to develop new antibacterial drugs, including those with novel mechanisms of action, the occurrence and dissemination of antibiotic resistance seem to be inevitable [1]. Under selection pressures, most pathogenic microorganisms can adapt and develop defenses against antibiotic attacks [2]. As the threat of antibiotic resistance spreads, adopting new strategies to complement or replace current antibiotics is becoming urgent [3,4].

One suggested approach to tackling antibiotic resistance is to optimize the accessibility of antibiotics at their designated sites of action using effective antibiotic dosage forms [3]. This concept, referred to as increased local bioavailability, plays a critical role in determining the clinical outcome [5,6]. By ensuring the sufficient exposure of antibiotics to harmful bacteria, they can be effectively eliminated before they have a chance to develop drug resistance. This strategy may also help prevent the formation of bacterial biofilms when they are treated with sub-lethal concentrations of antibiotics. Moreover, the use of suitable dosage forms not only enhances therapeutic efficacy, but also reduces the occurrence of dose-related side effects and the emergence of resistance, thereby extending the lifespan of existing antibiotics [7,8]. This approach is essential in the short term since the widespread availability of novel antibiotics for clinical use is unlikely to happen immediately [9].

In this context, we recently developed a nano-carrier system with the aim to improve the pharmacokinetics of antibiotics [10]. This formulation was prepared via the nano-precipitation method utilizing a biodegradable and highly biocompatible polylactic-co-glycolic acid polymer (PLGA). *Staphylococcus aureus*, a leading cause of a wide range of clinical infections, was selected as the representative model of the bacterial pathogen. To target staphylococcal infection sites, the surfaces of nano-particles were conjugated with anti-protein A antibodies, which have a high affinity for bacterial surfaces. The conjugation process was optimized to ensure the proper orientation of antibodies on the nano-particle surface, preserving their antigen-recognition ability [10,11]. In vitro experiments and pharmacokinetic studies conducted on infected mouse models provided evidence of the successful targeting of the nano-carrier system at the desired sites of infection. As proof of concept, a hydrophobic antibiotic, rifampicin, was loaded into the nano-carrier (nano-antibiotic) to examine the susceptibility of *S. aureus* to the formulation.

One of the key advantages of this strategy is that the nano-carrier formulation can be used with a diversity of antimicrobial agents, depending on the susceptibility of targeted bacteria [9]. In this study, we investigated the loading capacity of the targeted nano-carrier system using two fluoroquinolone antibiotics with different physicochemical properties, one hydrophile molecule, ciprofloxacin (CIP), and one amphiphile molecule, levofloxacin (LEV) [12,13]. 

Regarding pharmacology, both CIP and LEV are broad-spectrum antibiotics that are commonly used to treat a wide range of bacterial infections, including those caused by *S. aureus* [14]. CIP and LEV have relatively short average plasma elimination half-lives, typically lasting from around 6 to 8 h following single oral or intravenous administration. The maximum plasma concentration (C_max_) of both antibiotics is approximately 10 g/L [15,16]. Despite their proven effectiveness, several challenges limit the successful clinical use of CIP and LEV [17]. One of the major obstacles is the poor therapeutic concentrations of these antibiotics at the site of infection. For instance, in the case of CIP, only a small fraction (from 0.5% to 5%) of the administered dose actually enters the bloodstream after oral administration, and only about 10% of the circulating antibiotic reaches the site of infection [18]. Moreover, the frequent administration of these antibiotics is often accompanied by various side effects [19]. Thereby, the design and utilization of targeted formulations may offer significant advantages in improving drug bioavailability, reducing toxicity, minimizing dosing frequency, and enhancing patients’ adherence to the prescribed treatment regimen [20,21]. 

Despite their similar antibacterial activity against *S. aureus*, the distinct physicochemical properties of these fluoroquinolones may impact the formulation and loading method within the drug carrier [13,22]. In this study, we first optimized the drug-loading techniques for two antibiotics. Successful LEV- and CIP-loaded nano-carriers were further conjugated with anti-S. aureus antibodies. The interaction of these nano-antibiotics with *S. aureus* cells was evaluated via confocal microscopy. Conventional tests (MIC and MBC) were conducted to examine the antibacterial properties of the nano-antibiotic formulations. In particular, using the bioluminescent strain, *S. aureus* Xen29, we compared the efficacy of nano-antibiotics with their free form by measuring the amount of MBC and MBEC based on the bioluminescence technique. This new method addresses the limitations of evaluating the antibacterial efficacy of colloidal systems. Furthermore, a short-time killing assay of bacteria, mimicking the dynamic environment in the body, was employed to compare the efficacy of the nano-antibiotics with the free-form antibiotics. 

## 2. Results and Discussion

### 2.1. Nano-Antibiotic Preparation and Characterization

The present work aims to develop a targeted drug-delivery system in order to improve bioavailability and maximize the effectiveness of existing antibiotics. Firstly, ciprofloxacin (CIP) and levofloxacin (LEV), two representative fluoroquinolone antibiotics, were incorporated into PLGA-based nano-carriers. Nano-formulations were prepared using a nano-precipitation method [10]. Blank nano-particles (NP) that were unloaded with antibiotics had a hydrodynamic diameter of about 100 nm and a polydispersity index (PDI) of 0.1, exhibiting desirable properties for systemic administration [23]. 

**Figure 1:**
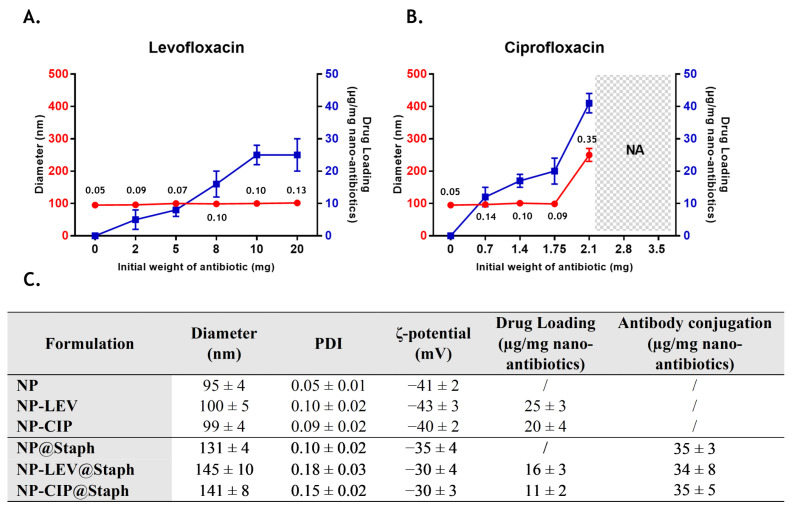
Characterization of nano-antibiotic formulations. Influence of the initial amount of levofloxacin (LEV) (**A**) and ciprofloxacin (**B**) on the particle size (red line), PDI (next to particle size) and drug loading (blue line) of nano-antibiotics; NA: data not determined due to the aggregation. (**C**) Main physicochemical characterization of un-modified nano-antibiotics and targeted nano-antibiotics. Data are presented as mean ± SD; n ≥ 3.

To accommodate the distinct physicochemical properties of the two antibiotic drugs, slight modifications were made to the nano-antibiotic preparation protocol [24,25]. Specifically, since LEV is soluble in both water and many organic solvents, it was directly integrated into the organic phase containing PLGA. In contrast, due to the poor solubility of CIP in organic solvents, the antibiotic was first dissolved in water at a saturated concentration (35 mg/mL). Subsequently, the CIP solution was poured into the organic phase containing PLGA. The resulting organic phase solutions (containing PLGA+LEV or PLGA+CIP) were then injected into an aqueous phase containing 0.5% Pluronic F68 to precipitate nano-antibiotics. The pH of the aqueous phase was maintained at pH 7, corresponding to the isoelectric point of both CIP and LEV, to prevent the significant diffusion of antibiotic molecules into the aqueous phase during nano-precipitation [12]. To optimize drug entrapment, various formulations were prepared with varying amounts of initial LEV (from 2 to 20 mg) and initial CIP (from 0.7 to 3.5 mg, which is equivalent to 20 to 100 µL of saturated CIP solution). The resulting nano-carrier formulations were assessed for their average size (Dh), PDI, and drug loading. PDI serves as an indicator of the size distribution quality of the nano-formulation. A narrow particle size distribution with a PDI value below 0.2 is highly desirable for drug delivery purposes [23]. The drug loading of the nano-antibiotic was expressed as μg of antibiotics per mg of the nano-formulation. A higher degree of drug loading is preferable as it reduces the amount of pharmaceutically inactive ingredients, thereby minimizing side effects and enhancing therapeutic efficiency [23]. Corresponding data for different formulations of nano-antibiotics are summarized in Figure 1A,B.

Concerning LEV-loaded formulations (Figure 1A), no significant change in particle size was observed when the initial amount of antibiotic was varied. However, the rate of antibiotic loading increased as the initial mass of LEV was raised, reaching the highest loading capacity of 25 µg/mg of nano-antibiotics from 10 mg of initial LEV mass. These obtained drug loadings are relatively low, which is likely due to the diffusion and re-distribution of LEV between the dispersed organic phase and the external aqueous phase during precipitation [26]. Nevertheless, this result correlates with previously reported data from similar systems involving PLGA nano-particles loaded with LEV [22]. In the subsequent experiment, the LEV-loaded nano-carrier formulation (NP-LEV) was produced using an initial LEV mass of 10 mg, aiming to achieve the maximum loading capacity.

Regarding CIP-loaded formulations (Figure 1B), the average size of the resulting nano-antibiotics remains unaffected by the presence of CIP up to an initial mass of 1.75 mg. However, when the initial quantity exceeds 2.1 mg, the average size increases significantly, accompanied by the formation of numerous aggregates during the nano-precipitation process (from 2.8 mg). This phenomenon could be attributed to the instability of CIP in the organic phase, leading to its precipitation when the initial amount of CIP is excessively high [27,28]. Like LEV, the amount of encapsulated CIP increases by the initial mass of CIP, reaching a drug loading total of 41 µg CIP/mg of nano-antibiotics. However, at this highest level of drug loading, nano-antibiotics exhibit heterogeneity in size (PDI > 0.2), and the mean size exceeds the desired range for systemic drug delivery (>200 nm) [23]. Consequently, an initial CIP concentration of 1.75 mg was fixed to maintain a stable size of CIP-loaded nano-antibiotics (NP-CIP) and ensure drug loading at the highest level.

To develop a carrier system capable of targeting *S. aureus* bacteria, we employed carbodiimide chemistry to conjugate anti-staphylococcal antibodies (@Staph) onto the surface of nano-antibiotics, NP-LEV and NP-CIP. The optimized conjugation protocol was used to ensure the controlled orientation of antibodies on the nano-antibiotics, maximizing their accessibility to antigen-binding sites [10]. The targeted nano-antibiotic formulations, referred to as NP-LEV@Staph and NP-CIP@Staph, were characterized in terms of their size, PDI, zeta potential, and drug loading, as summarized in Figure 1C. Following the grafting of antibodies onto the surface of nano-antibiotics, a decrease of 45% in drug loading for both types of formulation was observed, which is likely due to additional washing steps after grafting. Additionally, the conjugation process increased the size of nano-antibiotics to be approximately 40% larger than their unmodified counterparts, which was accompanied by a reduction in the surface charge (absolute value). A significant increase in size was observed in previous studies, suggesting that antibodies were successfully conjugated to nano-particles [29,30,31]. The amount of conjugated antibodies in the targeted nano-carriers was determined at approximately 35 µg antibodies per 1 mg nano-antibiotics, which aligns with previous reports [10,32]. 

In order to ensure that antibiotic loading did not influence the targeted bacterial properties of nano-antibiotic formulations, we assessed their interaction with *S. aureus* bacteria using fluorescence microscopy. For the visualization of nano-antibiotics, both the fluorescent DID dye and antibiotics were co-encapsulated in the same carrier. However, a significant increase in particle size was observed, along with system instability, which could potentially be attributed to unwanted interaction between the DID dye and the antibiotics. Consequently, antibiotic-free fluorescent nano-carriers (NP-DID and NP-DID@Staph) were prepared separately for fluorescence microscopy observation experiments. The encapsulation of DID on the nano-carriers did not significantly modify the characteristics of blank nano-carriers.

**Figure 2:**
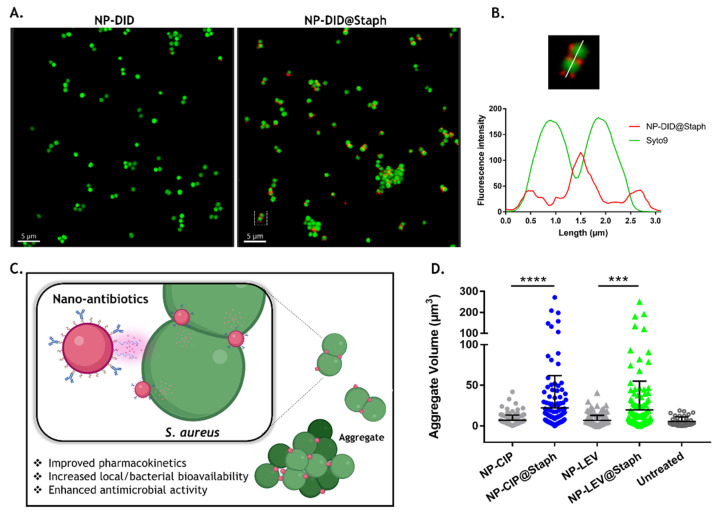
Interaction between nano-antibiotics and *S. aureus*. (**A**) Representative fluorescence images of *S. aureus* Xen29 after 10 min exposure with unmodified (NP-DID, left) or targeted nano-carriers (NP-DID@Staph, right); *S. aureus* cells were stained with Syto9 (green), and nano-carriers were pre-loaded with fluorescent DID dye (red). (**B**) Profile lines along a relevant ROI showing the distribution of NP-DID@Staph (red line) on the surface of two undivided *S. aureus* cells (green line). NP-DID@Staph is heterogeneously distributed on the cell surface and present at the cross-wall cell. (**C**) Schematic illustrates the binding of nano-antibiotics on the surface of *S. aureus* and the aggregation phenomenon, potentially improving the biodistribution of antibiotics. Then, the release of the encapsulated drug potentially leads to an efficient antibacterial effect. (**D**) Quantification of aggregate bacterial volume after 10 min exposure of un-modified nano-antibiotic (NP-LEV and NP-CIP) and targeted nano-antibiotic (NP-LEV@Staph and NP-CIP@Staph). The complementation of antibodies on the nano-antibiotic surface significantly increased the clumper aggregate volume. Statistical analyses were performed using the Mann-Whitney test, *** *p* <0.001, **** *p* < 0.0001.

Following 10 min incubation with NP-DID or NP-DID@Staph, the binding capacity of nano-carriers on the bacterial surface was visualized using fluorescence microscopy. Figure 2A (left) demonstrates that the control NP-DID, lacking conjugated antibodies, failed to adhere to the bacterial cells. In contrast, the fluorescent NP-DID@Staph nano-particles were detected at a high level of abundance around the bacterial surface (Figure 2A, right). Moreover, multiple focal planes revealed an uneven distribution of fluorescent NP-DiD@Staph on the bacterial surface. Notably, a higher presence of NP-DiD@Staph was observed at the division sites of incompletely separated cells (Figure 2B). 

Our findings align Schneewind’s report and support the hypothesis regarding the distribution of surface protein A in *S. aureus* bacteria [33]. Indeed, protein A deposition within the envelope of *S. aureus* occurs at discrete locations [34]. However, during proliferation, there is an increase in peptidoglycan and protein A at the cross wall that separates the dividing staphylococcal daughter cells. This increase results in a more diffused pattern of protein deposition, forming a ring-like structure of protein A that traverses areas of cell wall synthesis [33]. The extensive presence of protein A at this cross wall may be a crucial factor contributing to the significant adhesion of targeted nano-carriers.

Apart from adhering to the targeted bacteria, fluorescein-labeled NP-DID@Staph also exhibited the ability to bind bacteria together, resulting in the formation of aggregates with large volume sizes ranging from 10 to 300 μm^3^ (as shown in Figure 2A and illustrated in Figure 2C). It is worth noting that staphylococcal bacteria often remain incompletely separated on their cell wall, resembling clusters of grape-like cells in various growth conditions. However, our observation revealed that the presence of NP-DID@Staph amplified the size of these aggregates.

To investigate whether this phenomenon also presented on targeted nano-antibiotics (NP-CIP@Staph and NP-LEV@Staph), we conducted measurements of bacterial aggregate sizes in *S. aureus* cells following a 10 min of exposure to nano-antibiotics. To minimize the unwanted bactericidal effects, the concentration of nano-antibiotics was evaluated at sub-minimum inhibitory concentrations, which correspond to bacteriostatic levels. As a control, the treatment with NP-LEV and NP-CIP did not have any impact on the aggregation size of *S. aureus* (Figure 2D). However, as anticipated, the targeted nano-antibiotics induced the significant aggregation of *S. aureus* more compared to that of the untargeted counterparts. The results indirectly demonstrated the successful recognition and binding abilities of the two systems, NP-CIP@Staph and NP-LEV@Staph. The deposition of targeted nano-antibiotics on the surface of bacteria, coupled with the bridging aggregation of cells, may potentially enhance the distribution of nano-antibiotics within populations of bacterial pathogens [9,31]. Moreover, bacterial aggregation may facilitate efficient drug cargo release, concentrating it around the bacteria and reducing the off-target delivery of antibiotics (Figure 2C). 

### 2.2. In Vitro Antimicrobial Studies of Nano-Antibiotics

**Figure 3:**
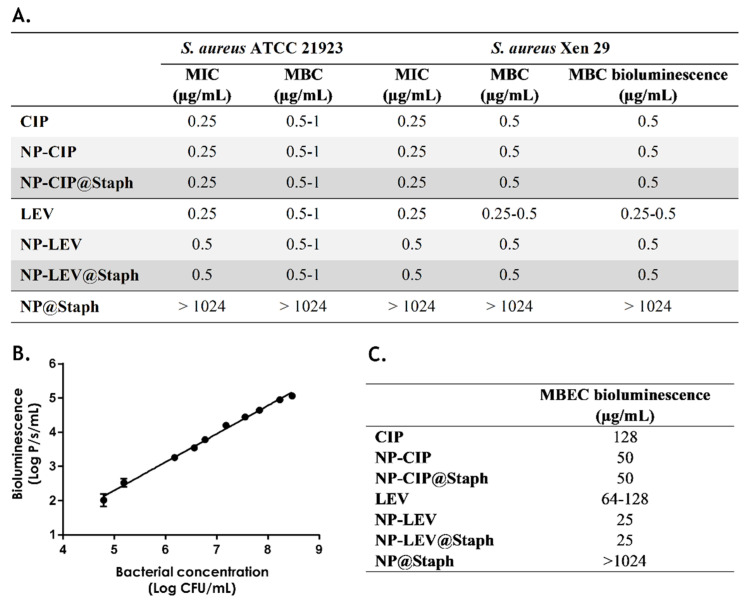
In vitro antimicrobial studies of nano-antibiotic formulations. (**A**) Minimum inhibitory concentration (MIC) and minimum bactericidal concentration (MBC) of free-form antibiotics and nano-antibiotics against *S. aureus* ATCC 21923 and Xen29; n ≥ 5. *(***B**) Correlation between bacterial density (CFU/mL) and bioluminescence intensity (P/s/mL); n = 3. (**C**) Bioluminescence-based minimum biofilm eradication concentration (MBEC) of free-form antibiotics and nano-antibiotics against *S. aureus* Xen29 biofilm; n ≥ 5.

In this section, we assessed the effectiveness of nano-antibiotics by determining their minimum inhibitory concentration (MIC) and Minimum bactericidal concentration (MBC). Our study focused on two strains of *S. aureus*: ATCC 25923 and Xen29. In their free form, both CIP and LEV exhibited similar MIC and MBC values across the two bacterial strains. Both antibiotics exhibited bactericidal activity, with the MBC values aligning with their MIC values (Figure 3A). 

However, when considering nano-antibiotics, we discovered that their MIC and MBC values were either equivalent to (in the case of NPs-CIP@Staph) or higher than (in the case of NP-LEV@Staph) those of their free-form antibiotics. This observation is consistent with numerous studies that have reported increased MIC and MBC values for nano-antibiotic formulations [9,28]. One possible explanation for this phenomenon is the incomplete release of antibiotics from nano-antibiotics, resulting in an insufficient number of free antibiotics that are able to effectively exert their action.

In parallel, we employed a real-time bioluminescence measurement technique to evaluate the antimicrobial activity of nano-antibiotics [35]. The bioluminescent *S. aureus* Xen29 strain was generated via integrating the luciferase operon from *P. luminescence* into the bacterial chromosome [36]. This integration allows bacteria to produce both the luciferase enzyme and its substrate, resulting in the generation of a bioluminescent signal during cellular metabolism. By monitoring bioluminescence, the microbial load can be rapidly assessed in a semi-quantitative manner. Additionally, variations in environmental conditions, such as antibiotic concentrations, can be immediately detected via changes in the total bioluminescence signal [10]. A correlation between the *S. aureus* Xen29 bacterial density and bioluminescence intensity was confirmed (Figure 3B). 

To examine the reliability of the bioluminescence technique, we first compared the bioluminescence-based MBC with the conventional MBC values of free-form antibiotics and nano-antibiotics. The bioluminescence-based MBC is considered to be the lowest concentration of antibiotics that completely inhibits bacterial bioluminescence after 24 h of treatment. Interestingly, the MBC values obtained using the bioluminescence technique were consistent with the conventional MBC values derived from bacterial counts after one day (Figure 3A). This suggests that the bioluminescence-based MBC estimation method could be reliable and yield similar results to the traditional approach based on bacterial counts.

We then assessed the minimum biofilm eradication concentration (MBEC) of free-form antibiotics and nano-antibiotics using the same bioluminescent technique. The bioluminescent-based MBEC represents the lowest concentration of antibiotics at which no bioluminescence is detected within the biofilm. In the initial step, an *S. aureus* biofilm was cultivated on a white 96-well plate for 24 h, and the formation of the biofilm was confirmed using the crystal violet test. Subsequently, free antibiotics and nano-antibiotics were added to the wells containing the biofilm. After 24 h exposure, the biofilm was washed twice with PBS and re-incubated with a fresh culture medium. Bioluminescence emitted by the biofilm was monitored after an additional 24 h. 

It was observed that the MBECs of free-form CIP and LEV were approximately 128 µg/mL, which is 256 times higher than their MBC (Figure 3C). This finding aligns with previous literature reports, indicating significant antibiotic tolerance in the biofilm state [37,38,39]. Surprisingly, both targeted and non-targeted nano-antibiotics exhibited a 2–5-fold reduction in MBEC values compared to those of their free-form antibiotics. This improved effectiveness of nano-antibiotics in eradicating biofilms has been previously investigated in our study and elsewhere [10,24,40]. The small size of nano-antibiotics, less than 200 nm, facilitates their penetration into the biofilm, leading to an increased antibiotic concentration in the innermost layers [40,41]. This hinders bacteria from adapting to the antibiotic concentration gradient, a common occurrence during free antibiotic therapy [42,43].

Although the bioluminescence method may not fully replace conventional MBC and MBEC determination, it offers several advantages when antibiotic-loaded nano-carriers are evaluated. Indeed, conventional antimicrobial activity assays are susceptible to errors and exhibit a high level of variability, especially in the presence of colloidal nano-particles [35]. In liquid media, the presence of nano-particles can cause turbidity, making it challenging to accurately measure bacterial growth. Moreover, the binding of nano-particles to the bacterial surface can effectively inhibit bacterial colony growth, while maintaining their metabolic activity. As a result, conventional MBC values may be overestimated when nano-antibiotic efficacy is assessed. 

In dealing with biofilms, the robust adhesion of bacterial cells within the biofilm matrix renders physical strategies, such as sonication, inadequate for achieving the complete dispersion of cells during the counting process [42,43,44]. In contrast, the direct measurement of bioluminescence within the biofilm for MBEC determination provides a more reliable assessment of the bacterial response to the tested compounds. Furthermore, bioluminescence-based measurements offer the advantage of saving time, allowing for the rapid screening of new antibiotic nano-delivery systems. This accelerated screening process enables researchers to evaluate the efficacy of different nano-carriers more efficiently.

### 2.3. Short-Time Killing Assay

**Figure 4:**
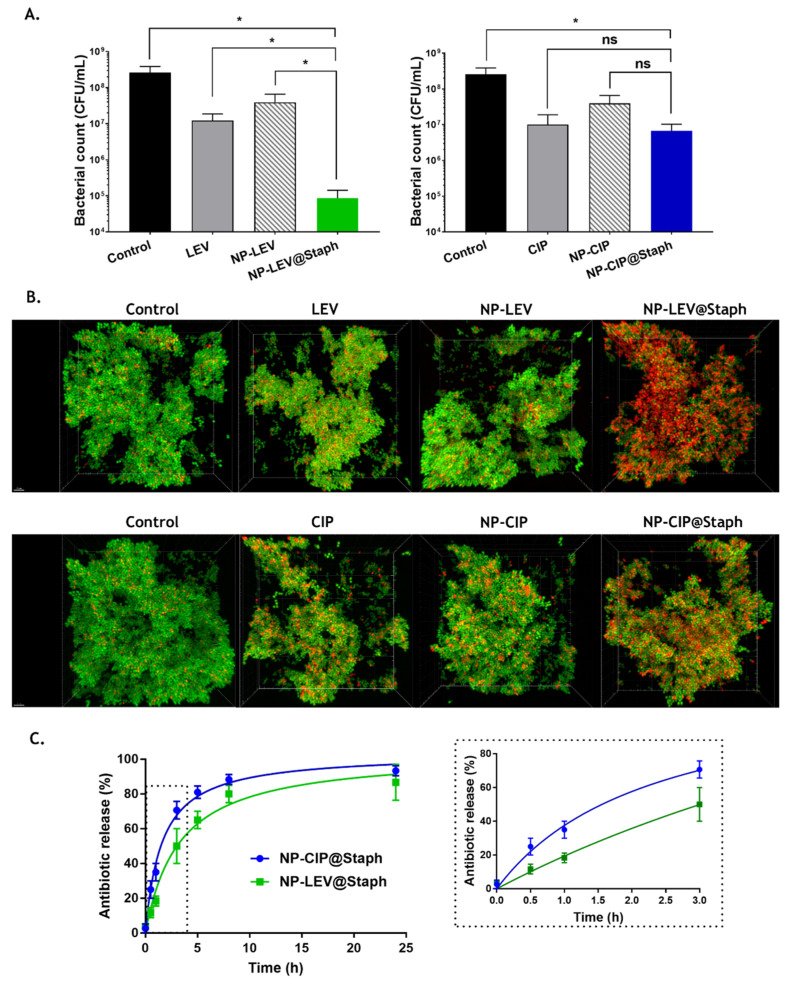
Antimicrobial efficacy of targeted nano-antibiotics in a short time-kill assay. (**A**) Bacterial killing effects of free-form and nano-antibiotic after a short-time incubation (10 min) with *S. aureus* Xen29; n ≥ 5. Statistical analyses were performed using the Mann-Whitney test, * *p* < 0.05. (**B**) Representative confocal fluorescence images of the bacterial pellet of *S. aureus* Xen29, showing the distribution of living bacterial cells (in green; stained with Syto 9) and dead cells (in red; stained with PI) following the antibiotic treatments. Scale bar: 30 µm. It should be noted that fluoroquinolones work via obstructing DNA replication, which effectively hinders bacterial reproduction without causing immediate cell destruction. Consequently, some cells might lack visible signs of PI inside immediately, despite their inability to form colonies following antibiotic treatment [48]. (**C**) In vitro drug release measurements of LEV and CIP from targeted nano-antibiotics; n = 3.

We were interested in seeing whether the targeted binding of nano-antibiotics to the bacteria had an impact on antimicrobial efficacy. Considering the limited residence time of nano-antibiotics at the infection site [45,46], bacteria cells were exposed to free-form antibiotics or nano-antibiotics at their C_max_ concentration (10 µg/mL) for a short interaction period of 10 min. Subsequently, the bacteria were washed and re-suspended in a fresh culture medium, followed by a five-hour incubation period. Surviving bacteria were counted on agar plates and assessed using fluorescent staining with a Live/Dead kit. As shown in Figure 4A, both the free-form antibiotics and nano-antibiotics led to a significant decrease in the bacterial count by 10- to 100-fold compared to that of the untreated group. Additionally, fluorescence imaging also demonstrated an increased presence of dead cells (red signal of PI labeling) in all treated groups. During this experiment, despite the relatively brief incubation period of antibiotics and bacteria interaction, the substantial reduction in bacterial cell viability underscored the post-antibiotic effect of fluoroquinolone antibiotics [47].

Notably, the untargeted nano-antibiotics (NPs-CIP and NP-LEV) groups exhibited lower efficacy than the free-form antibiotics groups did. Regarding the targeted nano-antibiotic treated groups, NP-LEV@Staph displayed remarkable effectiveness, resulting in a 100-fold reduction compared to that of the untreated group. Fluorescence imaging observation was also performed to correlate to these findings (Figure 4B). Conversely, the enhanced efficacy was not observed with the targeted NP-CIP@Staph, as there was no significant difference in bacterial concentrations compared to those treated with free-form CIP. 

As previously mentioned, since the primary mechanism of action for targeted nano-antibiotics relies on the release of antibiotic molecules at the infection site, we investigated the drug release profiles of the two formulations over time [49]. We sought to determine whether the differences in physicochemical properties between CIP and LEV could impact the drug release kinetics and antimicrobial efficacy of nano-antibiotics. As shown in Figure 4C, the drug release kinetics of the two formulations exhibited a biphasic pattern characterized by an initial burst release within the first 8 h. This initial burst release could be attributed to the diffusion of drug molecules initially entrapped near the surface of the nano-carrier matrix [50]. Following this, a slower release profile was observed, extending for up to 24 h during the study. When the drug release profiles of the two nano-antibiotics were compared, a notable difference was observed, particularly within the first 3 h (Figure 4C right). NP-CIP@Staph showed the rapid release of antibiotics, with approximately 25% of the drug payload being released within 30 min, which was twofold higher than it was for the NP-LEV@Staph formulation. This burst release could be attributed to the lower affinity of CIP for the polymer compared to that of LEV [51,52]. 

In the short-time killing experiment on bacteria, the repeated washing of bacteria (to simulate the kinetics of drug clearance in the body) could potentially enhance the removal of antibiotic cargo from nano-antibiotics, particularly in the case of CIP. This difference could account for the results of the nano-antibiotic efficacy experiment outlined earlier. While the targeted delivery capability of nano-antibiotics is important, our findings underscore the significance of controlling drug release to ensure the effectiveness of these formulations. The rapid release observed in NP-CIP@Staph may pose limitations on its clinical application. Ideally, a drug delivery system should effectively retain the loaded drug during transportation within the body and exhibit the rapid release of the active ingredient at target sites. 

It is important to acknowledge that these conclusions are based on our in vitro studies. While we have observed differences in the drug release profiles, we cannot exclude the possibility of variations in the number of NP-LEV@Staph and NP-CIP@Staph particles adhering to bacteria after the incubation period. Quantifying these targeted nano-antibiotics on the bacterial surface in future experiments is necessary to investigate this aspect further [53,54]. Moreover, conducting in vivo animal studies is essential to gain a more comprehensive understanding of the therapeutic effects of nano-antibiotics. In the treatment of infections, it is commonly recommended that someone administers a standard systemic dose of free antibiotics to ensure their bioequivalence and therapeutic effectiveness, even in the context of targeted therapy. In this case, we hypothesize that the observed burst release following the administration of nano-antibiotics could potentially provide a standardized systemic dose of antibiotics, ensuring their bioequivalence. Subsequently, the sustained release pattern, in conjunction with the targeting ability of nano-antibiotics, would result in a localized and high concentration of antibacterial agents at the infected site, thereby enhancing the treatment’s efficacy.

### 2.4. Cytotoxicity

**Figure 5:**
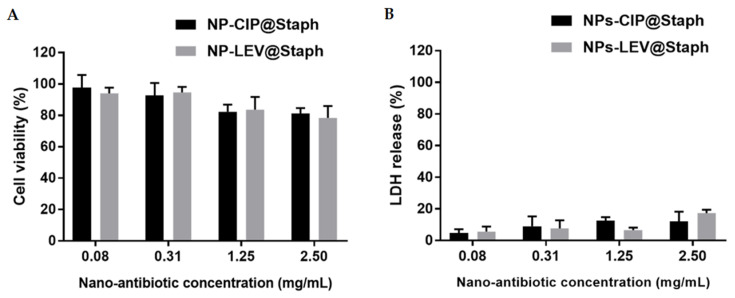
Cytotoxicity evaluation of nano-antibiotics. Estimated cell viability (**A**) and LDH release (**B**) of A549 cells after 24 h exposure to with NP-CIP@Staph and NP-LEV@Staph. Data are presented as mean + SD; n = 6.

Safety evaluation is essential when one is considering the clinical application of any novel drug dosage formulations. Here, we assessed the potential toxicity of NP-CIP@Staph and NP-LEV@Staph on alveolar epithelial lung A549 cells. Various nano-antibiotic concentrations ranging from 0.08 to 2.5 mg/mL were applied to the cells for 24 h. Cytotoxicity was evaluated in terms of mitochondrial activity (MTT test) and the measurement of released intracellular enzyme lactate dehydrogenase (LDH) as an indicator of membrane permeability [55]. The results in Figure 5 revealed that no significant toxicity was induced by the nano-antibiotics after 24 h incubation, even at high concentrations. The cell viability exceeded 80% in all the tested nano-antibiotic formulations, which indicates a good degree of biocompatibility with A549 cells. The evaluation of membrane integrity via LDH release also showed that the nano-antibiotics did not display cytotoxicity in the A549 cells within the tested concentration range. These results are consistent with previous studies on PLGA-based nano-formulations, demonstrating their safety and biocompatibility [56,57].

## 3. Materials and Methods

### 3.1. Materials, Bacterial Strains, and Growth Conditions

PLGA (RESOMER^®^ RG 502 H, 50:50 lactide glycolide, Mw = 13,200 g.mol^-1^, Đ = 2.5), Ciprofloxacin (CIP), Levofloxacin (LEV), Pluronic^®^ F-68, 4-Morpholineethanesulfonic acid (MES), 1-Ethyl-3-(3-dimethyl aminopropyl) carbodiimide (EDC), N-hydroxy succinimide (NHS), Anti-Protein A antibody (polyclonal antibody produced in rabbit), and D-trehalose (from *Saccharomyces cerevisiae*, 99%) were obtained from Sigma-Aldrich (Saint-Quentin-Fallavier, France). 1,1′-Dioctadecyl-3,3,3′,3′-tetramethylindodicarbocyanine, 4-chlorobenzene sulfonate salt (DID, 644/665 nm), Filmtracer™ LIVE/DEAD™ Biofilm Viability Kit, Pierce™ BCA Protein Assay Kit, 3-(4,5-Dimethylthiazol-2-yl)-2,5-diphenyltetrazolium bromide (MTT) and the CyQUANT™ fluorescence LDH Cytotoxicity Assay were obtained from Invitrogen^TM^ (ThermoFisher Scientific, Illkirch, France). All other chemicals were commercial products of analytical grade or higher and were purchased from Sigma-Aldrich (Saint-Quentin-Fallavier, France). Ultrapure water used for all experiments was obtained from a Milli-Q system (Millipore, Burlington, MA, USA).

In this study, two strains of *S. aureus*, ATCC 25923 and Xen29, were employed. The bioluminescent *S. aureus* Xen29 (Perkin Elmer, Waltham, MA, USA) is a derivative of the parental strain, *S. aureus* ATCC 12600, and harbors a modified *Photorhabdus luminescens* luxABCDE operon integrated within the chromosome. Bacteria were cultured overnight in brain heart infusion (BHI, Difco, Detroit, MI, USA) broth at 37 °C for 140 rpm. Subsequently, bacterial cells were collected and washed once with PBS buffer via centrifugation prior to conducting the experiments. The bacterial concentration was determined by measuring the optical density at 600 nm (OD600). Additionally, the viable bacterial count was assessed by plating serial dilutions onto BHI agar plates, followed by incubation at 37 °C for 24 h.

The relationship between bacterial density and bioluminescence was examined in this study. A culture of *S. aureus* Xen29 was incubated in a BHI medium at 37 °C for 140 rpm until it reached maximum growth after 7 h. Subsequently, the bacterial suspension was transferred to a flat-bottomed, white, 96-well plate. Multiple successive dilutions from this suspension were prepared. Bioluminescence measurements were then taken using a plate reader (Victor3, 1420 Multilabel Counter, Perkin Elmer, Beaconsfield, UK), followed by colony counts on BHI agar. Data were used to plot a graph illustrating the correlation between bioluminescence and colony-forming units (CFU).

### 3.2. Nano-Antibiotic Preparation

The antibiotic-loaded nano-carriers (nano-antibiotics) were obtained using the standard nano-precipitation method. Briefly, 10 mg of PLGA was dissolved in 1 mL of mixture acetone: ethanol (8:2 *v/v*). Due to its amphiphilic property, LEV was directly dissolved with the polymer in the organic mixture. Concerning CIP, the antibiotic was pre-dissolved in water (35 mg/mL) beforehand, and this solution was added to the organic phase containing the polymer under vigorous agitation. Organic phases were then quickly poured into 10 mL of an aqueous solution containing Pluronic F68 1% *w/v* under stirring (1200 rpm) to form nano-antibiotics. Samples were stirred at 300 rpm for at least 3 h at room temperature to allow organic solvent evaporation to occur. The resulting NPs were collected via centrifugation (50,000× *g* for 15 min at 4 °C, Beckman TL-100 Ultracentrifuge, Rotor TLA-100, Brea, CA, USA). 

The conjugation of the anti-S. aureus antibody to nano-antibiotics was performed using a two-step protocol. In the first step, the carboxylate functions on the surface of nano-antibiotics were activated using NHS carbodiimide chemistry. A dispersion of 1.5 mg of nano-antibiotics (equivalent to 0.1 μmol of carboxylate functions) was prepared in 1 mL of MES buffer (50 mM, pH 4.6) containing 1 μmol of EDC and 2 μmol of NHS. The mixture was incubated for 30 min and continuously, gently agitated at 4 °C. The activated nano-antibiotics were then recovered via centrifugation and resuspended in the same buffer at a concentration of 1.5 mg/mL. In the next step, 100 μg of anti-S. aureus antibodies (dissolved in 1 mL of MES buffer) was added to the suspension of pre-activated nano-antibiotics. The samples were incubated for 2 h at 4 °C and continuously, gently agitated to allow antibody conjugation to occur. Subsequently, nano-antibiotics were washed twice with PBS buffer (10 mM, pH 7.4) via centrifugation to remove any excess unconjugated antibodies. The amount of unconjugated antibodies in the supernatants was determined (Pierce BCA Protein Assay Kit (Thermo Fisher Scientific, Illkirch, France) to indirectly calculate the amount of antibody conjugated on the surface of the nano-antibiotics (in μg antibodies/mg nano-antibiotics). Finally, the nano-antibiotics were lyophilized for 24 h, with the addition of Trehalose as a cryoprotectant.

Fluorescent-labeled nano-carriers were produced by incorporating a lipophilic DID dye (Thermo Fisher Scientific, France) into the organic phase during the nano-carrier formulation. The optimal amount of DID was fixed at 0.5% *w/w* relative to the mass of the polymers.

### 3.3. Formulation Characterization

Particle size (Dh) and ζ-potential were measured with a Nanosizer Nano ZS instrument (Malvern, UK). The polydispersity index (PDI), obtained by the cumulants method, was used as an indicator of size distribution. All sample measurements were performed at 25 °C following dilution to a final concentration of 20 µg/mL of NPs in Milli-Q water.

To determine antibiotic loading, freeze-dried LEV-loaded nano-carriers without a cryoprotectant were dissolved in DMSO (1 mg/mL). The dispersion was then centrifuged, and the supernatant was analyzed for LEV concentration. The quantity of CIP encapsulated in the NPs was determined indirectly by measuring the quantity of the non-encapsulated antibiotic recovered in the supernatant after centrifugation and the washing of nano-antibiotics. The amounts of LEV and CIP were, respectively, quantified via absorbance measurement at 288 nm (levofloxacin) and fluorescence measurement at Ex/Em 275/450 nm (ciprofloxacin) using a plate reader (Flexstation 3, Molecular Devices, Winnersh, UK).

The release kinetics of antibiotics were evaluated in a sink condition [58]. To ensure the stability of antibiotics, release experiments were concluded after 24 h before obtaining the complete release profile. Antibiotic-loaded nano-carrier powders were dispersed in capped bottles containing 50 mL of PBS buffer (pH 7.4). The system was maintained at 37 °C with continuous stirring at 100 rpm. At specific time intervals, 500 μL of the medium was sampled, and the released antibiotic was separated from the nano-carriers using an Amicon^®^ Ultra (10 kDa, Millipore, Burlington, MA, USA). The released amount of antibiotic at each time point was determined, as described above, and the results were expressed as a percentage relative to the total antibiotic content loaded in the formulation.

### 3.4. Interaction of Nano-Antibiotics with Bacterial Cells

A total of 0.5 mL of the aliquot of bacteria was exposed to 0.5 mL of DID-loaded nano-carrier suspension (200 μg/mL in PBS) for 10 min at room temperature. Following the exposure period, bacteria were collected via low-speed centrifugation (3000× *g*) and washed twice with ice-cold PBS buffer to eliminate any unbound nano-carriers. The resulting bacterial pellet was then stained with 2.5 μM of Syto9 from the Filmtracer LIVE/DEAD biofilm viability kit (Molecular Probes-Invitrogen, Calsbad, CA, USA) for 15 min. Stained bacteria were observed using a Leica TCS SP8 confocal laser-scanning microscope (Leica Microsystems, Wetzlar, Germany). Initial experiments successfully determined the optimal protocol for generating bacterial suspensions consisting of individual, non-aggregated cells. Fluorescent images were captured using an oil immersion objective (×63) with a numerical aperture 1.4. Syto9-labeled cells and DID-loaded nano-carriers were excited at 488 nm and 650 nm wavelengths, respectively. The fluorescence emissions were collected using HyD detectors in counting mode, and the emission range was set between 500 and 540 nm for Syto9 and between 650 and 750 nm for the DID.

The same experimental setup was used to obtain nano-antibiotic formulations at a sub-minimum inhibitory concentration (0.5 MIC). The volume of formed bacterial aggregates (labeled by Syto9) after the treatment was quantified using Imaris software (Bitplane, Zürich, Switzerland). At least ten images from two independent experiments were analyzed.

### 3.5. Determination of Minimum Inhibitory Concentration (MIC) and Minimum Bactericidal Concentration (MBC)

The antimicrobial activity of nano-antibiotics was first evaluated in terms of the minimum inhibitory concentration (MIC) and minimum bactericidal concentration (MBC) against planktonic *S. aureus* ATCC 25923 and Xen29. The determination of MICs and MBCs was conducted using the standard microdilution method in 96-well microplates. In brief, overnight bacterial cultures were diluted in BHI medium to achieve a bacterial concentration of 10^6^ CFU/mL. Subsequently, 100 μL of either the antibiotic solution or an equivalent dose of nano-antibiotics was serially diluted in BHI, and 100 μL of the bacterial suspension was added to each well. The microplates were placed on a shaker and incubated at 37 °C for 24 h. The control groups consisted of wells containing either only the bacterial culture or BHI broth.

OD600 and bioluminescence measurements were then taken using a plate reader (Victor3, 1420 Multilabel Counter, Perkin Elmer). MIC was defined as the lowest concentration, which completely inhibits visible bacterial growth in the form of turbidity. The broth dilutions from MIC concentration were then streaked onto agar and incubated for 24 to 48 h. The MBC is the lowest-level broth dilution of antimicrobial that prevents the growth of the organism on the agar plate. The bioluminescence-based MBC is considered the lowest concentration of antibiotics that completely inhibits bacterial bioluminescence after 24 h of treatment.

### 3.6. Determination of Minimum Biofilm Eradication Concentration (MBEC) Based on Bioluminescence Measurement

Biofilms were initially grown on a white, 96-well plate for 24 h at 37 °C. Following that, the wells were washed with PBS and exposed to increasing concentrations of antimicrobial agents (free-form antibiotics and nano-antibiotics) for an additional 24 h at 37 °C. Subsequently, biofilms were washed twice with PBS buffer and incubated in a sterile BHI medium for 24 h at 37 °C. Bioluminescence measurements were then taken using a plate reader (Victor3, 1420 Multilabel Counter, Perkin Elmer, Beaconsfield, UK). The bioluminescence-based minimum biofilm eradication concentration (MBEC) represents the lowest concentration of antibiotics at which no bioluminescence is detected within the biofilm. Initial experiments determined that sonication did not impact the results obtained using the bioluminescence-based MBEC method. 

### 3.7. Short-Time Killing Assay of Bacteria

To assess the antibacterial effectiveness, a short-time killing assay model was utilized to mimic the dynamics of body clearance. A total of 500 μL of the culture of *S. aureus* Xen29 (2.10^7^ CFU/mL) was combined with 500 μL of either free-form antibiotics or encapsulated antibiotics to reach an antibiotic concentration of 10 μg/mL (equivalent to C_max_ of CIP and LEV). Following a 10 min exposure period, bacterial cells were separated from the free-form antibiotics or unbound nano-antibiotics via centrifugation (3000× *g*). The collected bacterial pellet was then washed twice with PBS buffer and was resuspended in 500 μL of fresh medium, following another round of incubation at 37 °C for 5 h. Subsequently, the samples were serially diluted and plated onto BHI agar, and viable cell counts were determined after 24 h at 37 °C. The results are reported as the mean log10 (CFU/mL). Additionally, treated bacteria were labeled using the Live/Dead kit according to the manufacturer’s protocol (Filmtracer LIVE/DEAD Biofilm Viability Kit, Molecular Probes-Invitrogen, Calsbad, CA, USA). The antibacterial efficacy of the nano-antibiotics was evaluated using confocal microscopy. Syto9 and PI fluorescent dyes were excited at wavelengths of 488 nm and 560 nm, respectively. The emitted fluorescence signals were captured between 500 and 540 nm for Syto9 and between 580 and 700 nm for PI.

### 3.8. In Vitro Cytotoxicity of Formulation

The cytotoxicity of nano-antibiotic formulations was assessed using a 3-[4,5-dimethylthiazol-2-yl]-3,5-diphenyl tetrazolium bromide (MTT) assay and Lactate dehydrogenase (LDH) test against the alveolar cell line A549. Cells were grown in DMEM supplemented with 10% (*v/v*) FBS, 100 IU/mL penicillin, and 100 µg/mL streptomycin. Firstly, A549 cells were seeded into flat-bottomed, 96-well plates at a density of 10^5^ cells/well and allowed to adhere under incubation at 37 °C and 5% CO2. The next day, the medium was replaced with 200 μL of fresh medium containing different concentrations of nano-antibiotics NP-LEV@Staph and NP-CIP@Staph (0.08–2.5 mg/mL) and further incubated for 24 h. Negative and positive controls were prepared by treating cells with media alone and 1% Triton-X, respectively. Following the treatment, the cell supernatant was collected and centrifuged (3700 rpm, 15 min). LDH released in the extracellular medium was quantified using the CyQUANT™ LDH Cytotoxicity Assay (ThermoFisher, Illkirch, France) according to the manufacturer’s instructions. The cell layer was washed twice with PBS and incubated with 100 μL of fresh media containing MTT reagent (0.5 mg/mL) for 2 h at 37 °C. The MTT solution was then replaced with 100 μL of DMSO to dissolve formazan crystals. Absorbance was measured at 570 nm. Cell viability (%) was then calculated as the ratio of the A570 from treated cells to A570 from control cells, which was considered to be 100 %. LDH release (%) was expressed as a percentage of the LDH release after nano-antibiotic exposure and the total LDH, which was measured after cell lysis using Triton X-100 (1 h, 37 °C). All measurements were performed in triplicate. 

### 3.9. Statistical Analysis

The experiments were performed at least in triplicate, and the results are reported as the mean ± standard deviation (SD). Statistical analysis was performed using the Mann–Whitney non-parametric test, with *p* < 0.05 being considered as statistically significant.

## 4. Conclusions

In the present study, we successfully encapsulated two fluoroquinolone antibiotics, ciprofloxacin and levofloxacin, within PLGA-based nano-carriers to develop nano-antibiotics. The surfaces of these nano-antibiotics were modified by conjugating anti-staphylococcal antibodies, which significantly improved their ability to recognize and bind to targeted bacteria. Our findings demonstrated that both untargeted and targeted nano-antibiotics increased the anti-biofilm efficacy more compared to that of the free-form antibiotics. The advantages of targeted delivery were particularly evident in the short-time killing assay of bacteria (dynamic environment). These targeted formulations may minimize the off-target effects on healthy tissues or the microbiome, while enhancing the bioavailability of antibiotics at the site of infection. However, it is crucial to note that the drug release kinetics of the formulation could influence the targeting effect. In future experiments, the consideration of incorporating controlled release strategies, such as stimuli-responsive drug release systems triggered by bacterial pathogens, should be taken into account to maximize the efficacy of antibiotic therapy. 

## Data Availability

Not applicable.
